# Spontaneously Formed Spheroids from Mouse Compact Bone-Derived Cells Retain Highly Potent Stem Cells with Enhanced Differentiation Capability

**DOI:** 10.1155/2019/8469012

**Published:** 2019-05-05

**Authors:** Kai Chen, Xianqi Li, Ni Li, Hongwei Dong, Yiming Zhang, Michiko Yoshizawa, Hideaki Kagami

**Affiliations:** ^1^Department of Hard Tissue Research, Graduate School of Oral Medicine, Matsumoto Dental University, Shiojiri 399-0781, Japan; ^2^Department of Oral and Maxillofacial Surgery, School of Dentistry, Matsumoto Dental University, Shiojiri 399-0781, Japan; ^3^Institute for Oral Science, Matsumoto Dental University, Shiojiri 399-0781, Japan; ^4^Department of Stomatology, Shanghai Tenth People's Hospital, Tongji University School of Medicine, Shanghai 200072, China; ^5^Department of General Medicine, IMSUT Hospital, Institute of Medical Science, University of Tokyo, Tokyo 108-8639, Japan

## Abstract

The results from our recent study showed the presence of two distinct spheroid-forming mechanisms, i.e., spontaneous and mechanical. In this study, we focused on the spontaneously formed spheroids, and the character of spontaneously formed spheroids from mouse compact bone-derived cells (CBDCs) was explored. Cells from (C57BL/6J) mouse leg bones were isolated, and compact bone-derived cells were cultured after enzymatic digestion. Spontaneous spheroid formation was achieved on a culture plate with specific water contact angle as reported. The expression levels of embryonic stem cell markers were analyzed using immunofluorescence and quantitative reverse transcription polymerase chain reaction. Then, the cells from spheroids were induced into osteogenic and neurogenic lineages. The spontaneously formed spheroids from CBDCs were positive for ES cell markers such as SSEA1, Sox2, Oct4, and Nanog. Additionally, the expressions of fucosyltransferase 4/FUT4 (SSEA1), Sox2, and Nanog were significantly higher than those in monolayer cultured cells. The gene expression of mesenchymal stem cell markers was almost identical in both spheroids and monolayer-cultured cells, but the expression of Sca-1 was higher in spheroids. Spheroid-derived cells showed significantly higher osteogenic and neurogenic marker expression than monolayer-cultured cells after induction. Spontaneously formed spheroids expressed stem cell markers and showed enhanced osteogenic and neurogenic differentiation capabilities than cells from the conventional monolayer culture, which supports the superior stemness.

## 1. Introduction

Somatic stem cells have a great potential for use in tissue repair and regeneration. Among them, mesenchymal stem cells (MSCs) have been widely used not only for basic research but also for clinical applications such as bone tissue engineering [1, 2]. Two-dimensional (2D) culture of adherent cells has been used as a standard technique for the in vitro expansion of MSCs, which is a relatively easy and generally accepted protocol [3, 4]. However, some reports have indicated the immediate loss of characteristic features of stem cells during culture, such as homing ability, replication capability, colony-forming efficiency, and differentiation capability [5–9]. To overcome these shortcomings, the potential of novel cell culture protocols has been explored [10–15].

One important breakthrough of somatic stem cell culture was the discovery of a floating culture, which was first reported for neural stem cells [16]. The presence of neural stem cells has been questioned for the long term. However, they were isolated from the embryonic brain using this novel culture protocol, which enabled selective, elongated survival and expansion of neural stem cells as spheroids [16–19]. Thereafter, this technique has been applied to the selective culture of various somatic stem cells, including mesenchymal stem cells [20–23]. Compared with traditional 2D cell culture, spheroids are a form of three-dimensional (3D) culture and are regarded for their ability to replicate the physiological environment for cells, thus better preserving the characteristics of somatic stem cells [24, 25]. The limitation of 3D culture includes the limited growth for mesenchymal stem cells [26] and low culture efficiency when the spheroid formed spontaneously [27].

There are several different approaches to generate spheroids (e.g., spinner flask method, liquid overlay method, and hanging drop method) [28, 29]. However, the differences among spheroids, obtained from different culture protocols, have yet to be shown. In this study, spheroid formation was achieved under static conditions on a plate with a specific water contact angle, which is around 90°. Because spheroid formation with this method occurs spontaneously, we designate this type of spheroid as a spontaneously formed spheroid [27]. Although the difference between spontaneously formed spheroids and mechanically formed spheroids (such as the spinner flask method) is not well known, spontaneously formed spheroids ideally consist of a purer stem cell population because spheroid formation starts from stem cells only, which possess the ability to proliferate enough to form spheroids. On the other hand, mechanically formed spheroids may contain various types of cells due to the forced aggregation of surrounding cells [27, 30]. However, most of the reported studies did not pay attention on the difference of those two methods, and in particular, the character of spontaneously formed spheroids with mesenchymal stem cells has not been well understood.

The most well studied and widely used cell source for mesenchymal stem cells is bone marrow mesenchymal stem cells (BMMSCs). However, some recent reports have shown that compact bone-derived cells (CBDCs) are a superior cell source compared with BMMSCs because CBDCs possess a higher proliferation and pluripotent differentiation capability [31–36]. In this study, we focused on spontaneously formed spheroids from CBDCs to characterize their potential as a somatic stem cell source. To our knowledge, this is the first report on spontaneously formed spheroids and spheroid-forming cells from mouse CBDCs.

## 2. Materials and Methods

All procedures for experiments in this study were performed in accordance with the guidelines laid down by the National Institutes of Health (NIH) in the USA, regarding the care and use of animals for experimental procedures, and approved by the Matsumoto Dental University Committee on Intramural Animal Use (No. 289).

### 2.1. Preparation of Mouse CBDCs

The cultivation protocol for CBDCs was conducted according to the protocol in our previous publication with some modifications [31]. Briefly, male C57BL/6J mice (3 weeks old, SLC Japan, Hamamatsu, Japan) were sacrificed with an overdose of anesthesia. The femurs and tibiae were disconnected from the trunk, and soft tissues were removed from the bone surface thoroughly. Epiphyses were cut, and bone marrow was flushed out using a syringe and 27-gauge needle with culture medium consisting of *α*-minimum essential medium with glutamine and phenol red (*α*-MEM, Wako Pure Chemical Industries Ltd., Osaka, Japan), supplemented with 1% penicillin-streptomycin-amphotericin B solution (Biological Industries Israel Beit Haemek Ltd., Kibbutz Beit Haemek, Israel). After the bone color became pale, the bones were placed in phosphate-buffered saline (PBS; Wako Pure Chemical Industries Ltd., Osaka, Japan) and were carefully cut into 1~2 mm fragments with scissors. Then, the bone chips were transferred into a 50 ml centrifuge tube containing 20 ml of PBS with 0.25% collagenase (Wako Pure Chemical Industries Ltd., Osaka, Japan) and 20% fetal bovine serum (FBS; Biowest, France). The tube was placed in a shaking incubator at 37°C with a shaking speed of 90 rpm. After 45 minutes of incubation, the cells were collected and transferred to another tube through a 40 *μ*m cell strainer (Falcon®, Corning, NY, USA). The tube was centrifuged for 5 minutes at 300 g at 4°C. The supernatant was removed, and the cell pellet was gently resuspended in *α*-MEM supplemented with 10% FBS, 1% penicillin-streptomycin-amphotericin solution, and 10 ng/ml recombinant human basic-fibroblast growth factor (bFGF; PeproTech, Rocky Hill, NJ, USA), which were used as the basic culture medium. The cell suspension was seeded into a culture dish (Falcon®, Corning, USA) at a density of 5.5 × 10^5^/cm^2^. Bone chips were collected and placed in a 30 × 15 mm cell culture dish with 2 ml of basal culture medium to collect additional cells. The primary cells were cultured at 37°C in a 5% CO_2_ humidified incubator. The medium was changed every three days. When the cells reached 70-80% confluence, the cells were detached with 0.25% trypsin-EDTA (Gibco: Life Technologies, Carlsbad, CA, USA) and subcultured in a new culture dish at a density of 1.5 × 10^4^ cells/cm^2^ until subconfluent.

### 2.2. CBDC Spheroid Formation

The method of spheroid formation was conducted according to the protocol in our previous publication [27]. Briefly, passage 2 CBDCs were resuspended in basic culture medium and transferred to a 55 × 17 mm low-adhesion culture dish (AS ONE, Osaka, Japan) at a density of 1.5 × 10^4^ cells/cm^2^ for spheroid formation, incubated at 37°C in a 5% CO_2_ humidified incubator. The density was optimized in our preliminary experiments (data not shown). The size and number of spheroids were observed using an inverted microscope (Olympus IX70, Olympus Optical Co. Ltd., Tokyo, Japan) at 12, 24, and 72 hours. At each observation time point, the size of spheroids was measured using 6 randomly selected fields (100x magnification) of a culture plate in 5 independent experiments. The photomicrographs were taken and used to measure the diameters of spheroids using the Olympus cellSens Standard 1.15 software. The number of spheroids was counted using a phase-contrast microscope at 40x magnification, which covered the entire plate.

### 2.3. Osteogenic and Neurogenic Induction of CBDCs

After 24 hours of spheroid formation, the spheroids were transferred into new conventional culture dishes to allow the spheroids to attach and spread on the bottom of the culture dish to grow as a monolayer. When CBDCs in monolayer culture or spheroids reached 50-60% confluence, the basic culture medium was replaced with osteogenic induction medium (basic culture medium, supplemented with 100 nM dexamethasone (Sigma-Aldrich, St. Louis, MO, USA), 50 *μ*M L-ascorbic acid phosphate (Wako Pure Chemical Industries, Ltd.), and 10 mM glycerol phosphate disodium salt hydrate (Sigma-Aldrich)) or neurogenic induction medium (basic culture medium, supplemented with 50 ng/ml recombinant nerve growth factor, 50 ng/ml recombinant brain-derived neurotrophic factor, and 10 ng/ml recombinant NT-3 (all three reagents from PeproTech, Rock Hill, NJ, USA)). During the induction process, the media were changed every two days.

### 2.4. Alkaline Phosphatase (ALP) Activity Assay

After 7 days of osteogenic induction, ALP activity was measured to confirm osteogenic induction. Noninduced cells were used as a control, which were continuously cultured in basic culture medium. An enzymatic assay (cell counting kit-8 (CCK-8); Dojindo Laboratories, Kumamoto, Japan) and p-nitrophenyl phosphate (SIGMAFAST^TM^ p-Nitrophenyl Phosphate Tablet; Sigma-Aldrich Co. LLC.) were used to evaluate cell proliferation and ALP activity according to the manufacturer's instructions. Formazan was measured at 450 nm, and p-nitrophenyl phosphate was quantified at 405 nm using an iMark™ Microplate Absorbance Reader (Bio-Rad Laboratories, Hercules, CA, USA).

### 2.5. Immunofluorescence Microscopy

Immunofluorescence staining was performed with embryonic stem cell markers. Spheroids were collected 24 hours after seeding to the low-adhesion plate and solidified in iPGell (Genostaff, Tokyo, Japan) according to the manufacturer's instructions, fixed with 4% paraformaldehyde in phosphate buffer, embedded in paraffin, and sectioned at a thickness of 8 *μ*m, as previously described [31]. Sections were permeabilized and blocked with 5% BSA, 5% goat serum, and 0.5% Triton X-100 in PBS. After incubation with primary antibodies overnight at 4°C, the sections were washed with PBS three times, followed by incubation with the respective secondary antibodies for 2 hours. Nuclei were counterstained with 4′,6-diamidino-2-phenylindole solution (Fluoroshield Mounting Medium with DAPI, ab104139, Abcam) for 30 min.

To confirm neurogenic induction, immunofluorescence staining for neural cell markers was performed after 14 days of induction. CBDCs from monolayer culture and spheroids were fixed with 4% paraformaldehyde in phosphate buffer for 20 minutes at room temperature followed by washing three times with PBS. The cells were treated with 5% BSA, 5% goat serum, and 0.5% Triton X-100 in PBS for 25 minutes at room temperature to permeate and block nonspecific binding of the antibodies. Primary antibodies were incubated with cells overnight at 4°C. After rinsing three times with PBS, the cells were incubated with the respective secondary antibodies for 2 hours at room temperature in dark and then washed three times with PBS. Nuclei were counterstained with DAPI for 30 min. The antibodies used are summarized in Table 1.

All fluorescent imaging was taken with a fluorescence microscope (Keyence BZ-X710, Keyence, Osaka, Japan) with 20x or 40x objective magnification. Cells incubated with secondary antibodies, without primary antibody incubation, served as a negative control.

### 2.6. RNA Extraction and qRT-PCR

qRT-PCR was performed to determine the expression levels of stem cell markers, osteogenic and neurogenic markers in spheroids, and CBDCs from spheroids or monolayer culture. Briefly, total RNA was extracted using the TRIzol reagent (Ambion®; Life Technologies, Carlsbad, CA, USA). After quantification of total RNA with a spectrophotometer (NanoDrop® ND-1000, Thermo Fisher Scientific, Waltham, MA, USA), RNA samples were reverse transcribed into complementary DNA (cDNA) using oligo (dT)12–18 primers (Life Technologies), dNTPs (Toyobo Co. Ltd., Osaka, Japan), and ReverTra Ace® (Toyobo Co. Ltd.) according to the manufacturer's instructions. qRT-PCR was performed in a thermal cycler (Thermal Cycler Dice Real Time System II TP-900, Takara Bio, Japan) using the SYBR Premix Ex TaqII reagent (Takara Bio, Kusatsu, Japan) according to the manufacturer's protocol. Primer sets (Sigma-Aldrich Co.) used for the PCR experiment are listed in Table 2.

### 2.7. Statistical Analyses

The results are presented as the means ± standard error of the means (SEM). Statistical analyses were conducted using Student's *t*-test between two groups. A *P* value of less than 0.05 was considered statistically significant.

## 3. Results

### 3.1. The Generation of Spontaneous Spheroids from CBDCs

**Figure 1:**
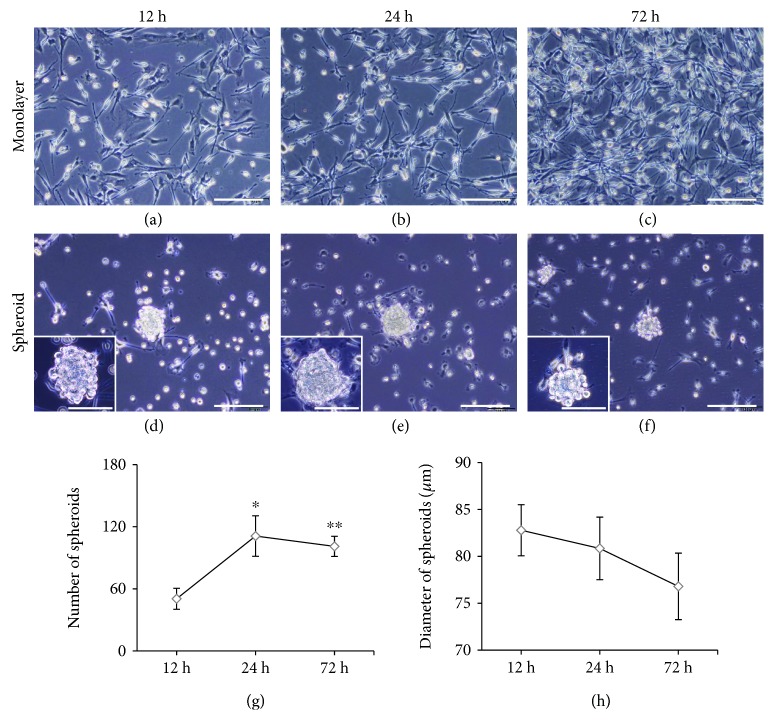
Spontaneous spheroid formation of CBDCs. On a conventional monolayer culture plate, adhered CBDCs showed fibroblast-like morphology and stable growth (a–c). On spheroid-forming plates, CBDCs began to form spheroids at 12 hours (d) and were maintained during the observation period (e and f). The number of spheroids peaked at 24 hours and then plateaued. The change in spheroid number between 24 hours and 72 hours was not statistically significant but was significant between 12 hours and 24 hours (*P* < 0.05) and also between 12 hours and 72 hours (*P* < 0.01), *N* = 5 (g). The average diameter of spheroids decreased gradually during the time course without statistically significant changes, *N* = 30 (h). Scale bars = 100 *μ*m. Data are represented as the mean ± SEM. ^∗^ *P* < 0.05 and ^∗∗^ *P* < 0.01.

When mouse CBDCs were seeded into a conventional plastic culture dish at passage 2, the cells adhered to the tissue culture plastic and showed fibroblast-like morphology after 12 hours (Figure 1(a)). The growth of CBDCs was stable, and the cell density increased after 24 hours (Figure 1(b)) and nearly reached confluence after 72 hours (Figure 1(c)). At 12 hours after seeding onto the low-adhesion culture plate, CBDCs began to form multicellular aggregates, which gradually became spheroids (Figure 1(d)). The spheroids were maintained during the observation period (Figure 1(e) and (f)). There were some spheroids that reattached on the dish and lost their spheroid morphology. However, no obvious cell death in spheroids was observed. The number of spheroids increased from 12 to 24 hours after cell seeding. The number of spheroids at 24 hours was significantly larger than that at 12 hours (*P* < 0.05). Then, the number of spheroids plateaued (Figure 1(g)). The average diameter of spheroids decreased gradually over time, but the difference was not significant (Figure 1(h)).

### 3.2. The Expression of Stem Cell Markers in Spheroids

**Figure 2:**
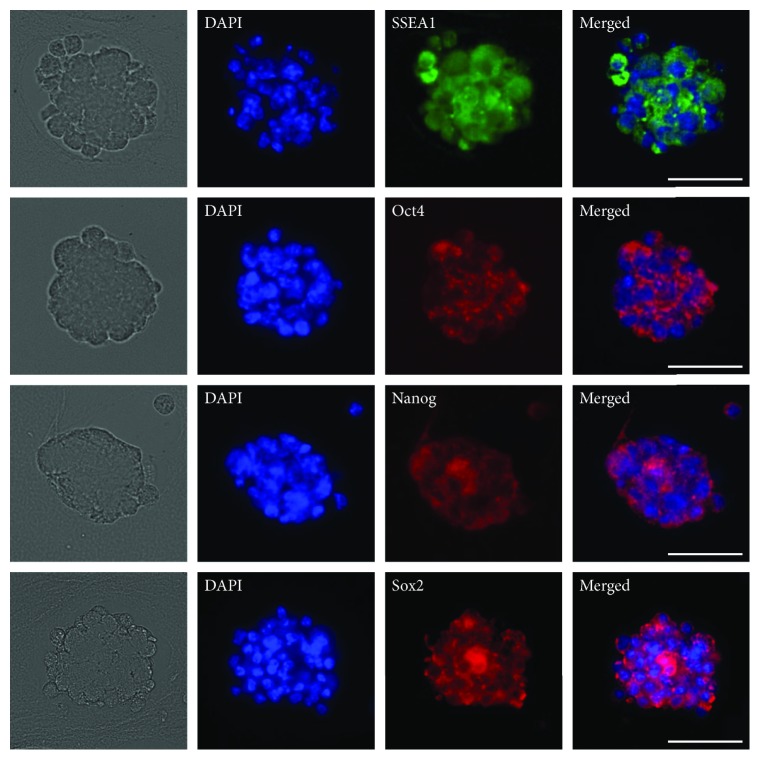
Immunofluorescence staining of spontaneously formed spheroids. CBDCs were cultured in spheroid-forming conditions for 24 h, and paraffin sections of spheroids were immunostained with primary antibodies specific for SSEA1, Oct4, Nanog, and Sox2 (corresponding to the first horizontal to the fourth horizontal). The positive reaction was distributed among almost all spheroid-forming cells. DAPI (4′,6-diamidino-2-phenylindole) was used for nuclear staining. Scale bars = 50 *μ*m.

The immunofluorescence results showed that the spheroids were positive for embryonic stem cell (ES cell) markers such as SSEA1, Oct4, Nanog, and Sox2, and the staining was exclusive to spheroid-forming cells. Positive cells were observed in almost all spheroids and evenly distributed for all examined ES cell markers (Figure 2).

**Figure 3:**
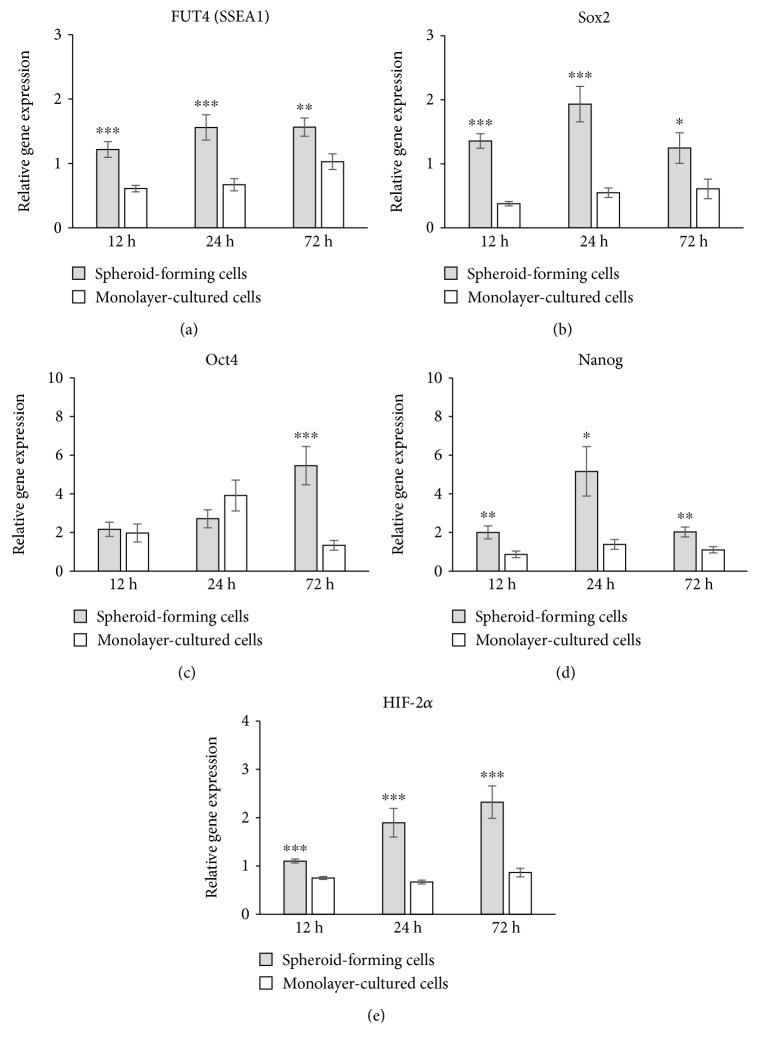
The expression of stem cell markers and HIF in spheroid-forming cells and monolayer-cultured cells. The relative expression of FUT4 (SSEA1) (a), Sox2 (b), Nanog (d), and HIF-2*α* (e) was significantly higher in spheroid-forming cells at any time point examined. The expression of Oct4 (c) in spheroids was significantly higher than that of monolayer-cultured cells at 72 hours. Data are represented as the mean ± SEM. (a and b) *N* = 5. (c) *N* = 4. (d) *N* = 6. (e) *N* = 3. ^∗^ *P* < 0.05, ^∗∗^ *P* < 0.01, and ^∗∗∗^ *P* < 0.001.

The results from qRT-PCR showed that the relative expression of FUT4 (SSEA1) (Figure 3(a)), Sox2 (Figure 3(b)), and Nanog (Figure 3(d)) was significantly higher in spheroids at any time point examined. The expression of Oct4 in spheroids was significantly higher than that of monolayer-cultured cells at 72 hours (Figure 3(c)). The expressions of all those ES cell markers were detected up to 120 hours (data not shown). The expression of HIF-2*α* in spheroids was significantly higher than that of monolayer-cultured cells at 12, 24, and 72 hours (Figure 3(e)).

**Figure 4:**
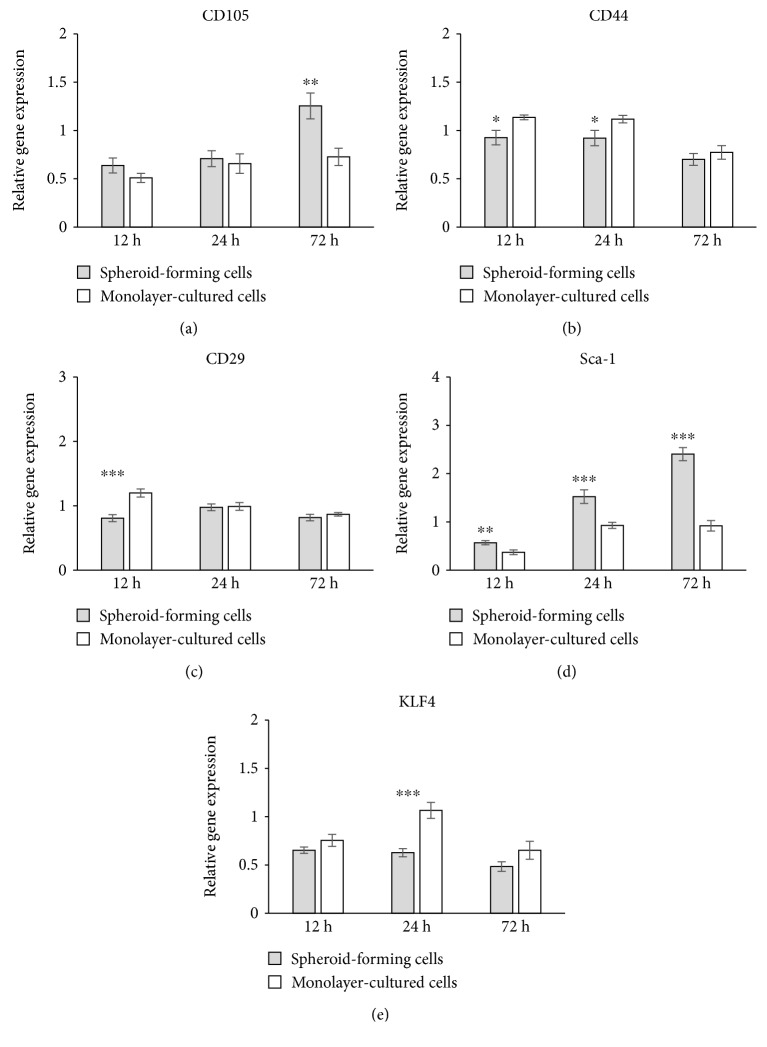
The expression of MSC markers in spheroid-forming cells and monolayer-cultured cells. The relative expression of CD105 (a), CD44 (b), CD29 (c), and KLF4 (e) was at close levels between spheroid-forming cells and monolayer-cultured cells, except for the individual observation time point. The relative expression of Sca-1 (d) showed a higher expression in spheroids than monolayer-cultured cells at all time points examined. Data are represented as the mean ± SEM. (a, d, and e) *N* = 4. (b and c) *N* = 3. ^∗^ *P* < 0.05, ^∗∗^ *P* < 0.01, and ^∗∗∗^ *P* < 0.001.

On the other hand, the expressions of MSC markers such as CD105, CD44, CD29, and KLF4 were almost identical between spheroids and monolayer-cultured cells (Figures 4(a)–4(c) and 4(e)), except for Sca-1, which showed a higher expression in spheroids than monolayer-cultured cells at all time points examined (Figure 4(d)).

### 3.3. Osteogenic Induction

**Figure 5:**
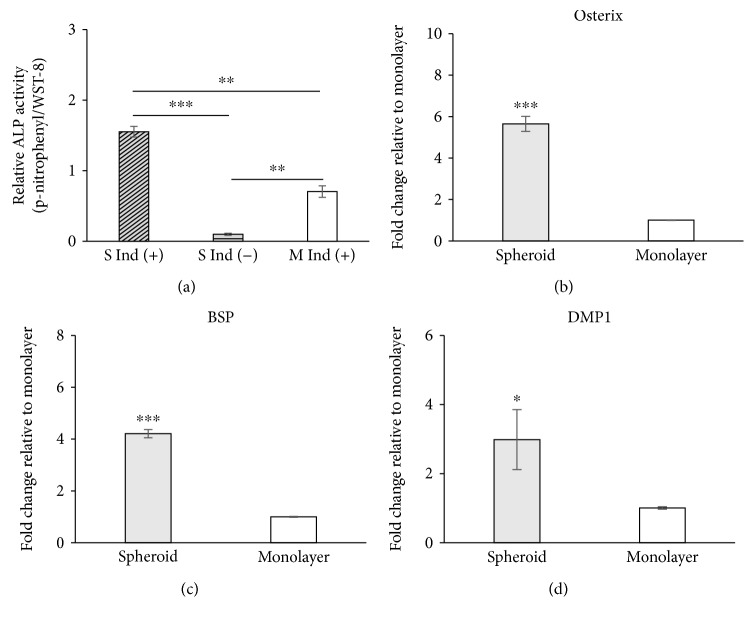
Osteogenic capability of spheroids. Monolayer-cultured CBDCs and spheroid-derived cells were incubated with osteogenic induction medium for 7 days. ALP assay data showed that induced spheroid-derived cells have significantly increased ALP activity compared with induced monolayer cells (a). qRT-PCR data showed that induced spheroid-derived cells expressed higher levels of osteogenic-related genes, such as osterix, BSP, and DMP1, with statistical significance (b–d). Data are represented as the mean ± SEM. ALP assay, *N* = 3; qRT-PCR, *N* = 3. ^∗^ *P* < 0.05, ^∗∗^ *P* < 0.01, and ^∗∗∗^ *P* < 0.001.

An ALP assay and qRT-PCR were performed to confirm the osteogenic induction at day 7. ALP activity was significantly higher in the induced groups than in the noninduced group for both monolayer and spheroid-derived cells (Figure 5(a)). The ALP activity of induced spheroid-derived cells was significantly higher than that of induced monolayer-cultured cells (*P* < 0.01).

The relative osteogenic marker gene expression levels were analyzed using qRT-PCR. The relative expression level of osterix in spheroid-derived cells was 5.65-fold higher than that in monolayer-cultured cells (Figure 5(b)). Similarly, the expression levels of BSP and DMP1 were higher than those in monolayer-cultured cells, and the differences were 4.21-fold and 2.98-fold greater, respectively (Figures 5(c) and 5(d)).

### 3.4. Neurogenic Induction *In Vitro*

**Figure 6:**
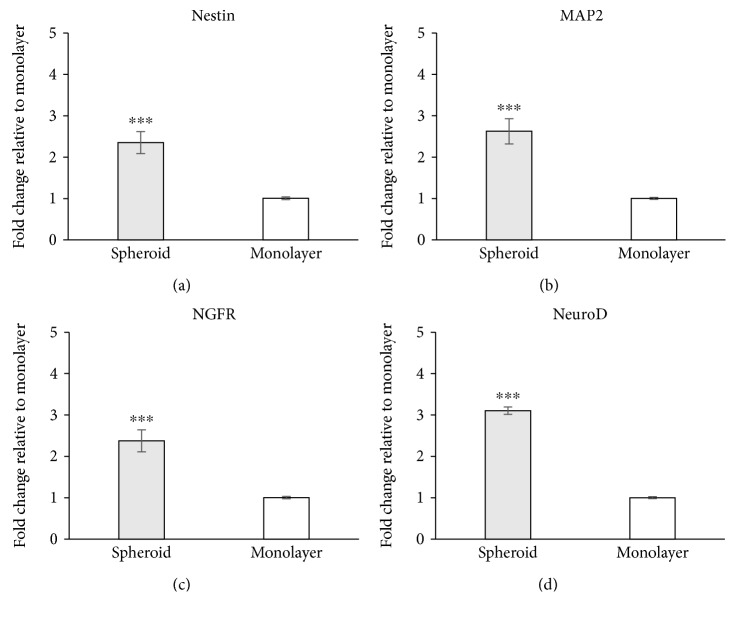
Expression of neurogenic-related genes in induced spheroid-derived and monolayer-cultured cells. Spheroid-derived cells and monolayer cells were cultured with neurogenic induction medium for 2 weeks. The expression of Nestin (a), MAP2 (b), NGFR (c), and NeuroD (d) in spheroid-derived cells was more than 2-fold higher than that in monolayer-cultured cells. Data are represented as the mean ± SEM, *N* = 4, and ^∗∗∗^ *P* < 0.001.

The qRT-PCR results showed that spheroid-derived cells had a significantly higher Nestin expression (2.35-fold) after 2 weeks of neurogenic induction (Figure 6(a)). The expression of MAP2 and NGRF in induced spheroid-derived cells was 2.62- and 2.38-fold higher than that in induced monolayer cells, respectively (Figures 6(b) and 6(c)). The expression of NeuroD in induced spheroid-derived cells was also significantly higher (3.10-fold) than that in induced monolayer cells (Figure 6(d)).

**Figure 7:**
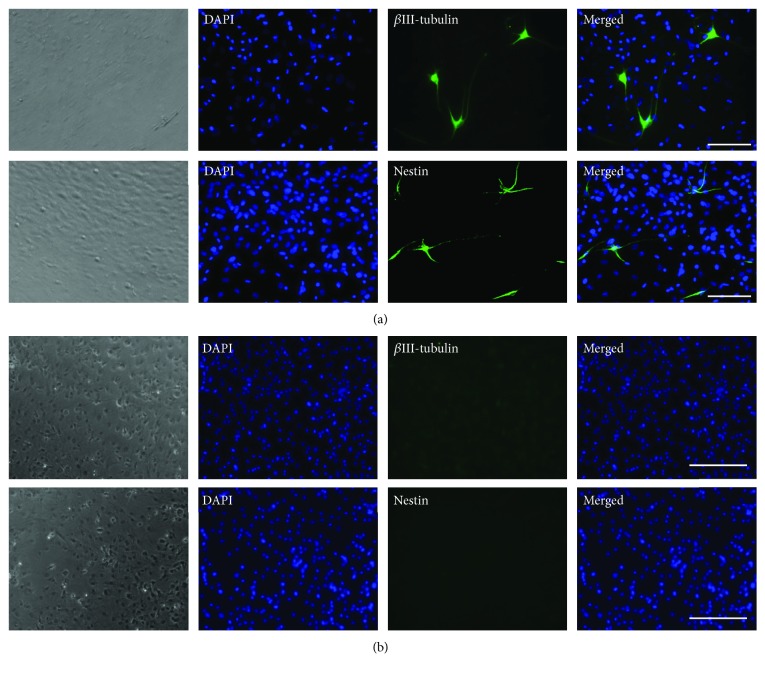
Immunofluorescence staining of neurogenic-induced spheroid-derived and monolayer-cultured CBDCs. After 2 weeks of neurogenic induction, spheroid-derived cells and monolayer-cultured cells were confirmed by immunofluorescence staining. The expression of Nestin and *β*III-tubulin was observed with neural cell-like morphology only in spheroid-derived cells (a). In contrast, monolayer-cultured cells showed no positive staining for both Nestin and *β*III-tubulin (b). DAPI (4′,6-diamidino-2-phenylindole) was used for nuclear staining. Scale bars = 50 *μ*m.

Furthermore, immunocytochemical analysis was performed to examine the distribution of the neural cell marker proteins in induced spheroid-derived cells and monolayer-cultured cells. Immunofluorescent images showed that the expression of Nestin and *β*III-tubulin was observed with neuronal-like morphology only in spheroid-derived cells, though the ratio of positive cells was relatively small (0.13% and 0.053% for Nestin and *β*III-tubulin, respectively) (Figure 7(a)). In contrast, monolayer-cultured cells showed no positive staining for either Nestin or *β*III-tubulin (Figure 7(b)).

## 4. Discussion

Spheroid formation from CBDCs was observed as early as 12 hours and peaked at 24 hours. Compared with spontaneous spheroid formation from neural cells and skin-derived cells, it occurs relatively early. Because our spheroid-forming method utilizes low-adhesion culture dishes, the early spheroid formation from CBDCs might reflect the relatively low adherence of spheroid-forming cells (possibly somatic stem cells) from CBDCs compared with those from neural- or skin-derived cells. To support this idea, the average size of spheroids from CBDCs (80.14 ± 19.27 micrometers in diameter) was smaller than that from skin-derived cells (approximately 100 micrometers). The spheroid diameter decreased over time. This finding might be due to the condensation of spheroid-forming cell aggregates, which was also observed in spheroids from other cell sources, such as periodontal ligament-derived cells [37].

To the best of our knowledge, this is the first study showing the expression of ES cell markers in CBDCs. Spheroids from CBDCs are positive for SSEA1, Oct4, Nanog, and Sox2, which suggests that the spheroid-forming cells from CBDCs are highly potent stem cells. The qRT-PCR results confirmed this result, and the expression of stemness markers such as FUT4, which encodes the SSEA1, Nanog, and Sox2 in spheroids, was significantly higher in spheroid-forming cells than in monolayer cells. At present, it is not fully understood why spheroid formation can switch on the expression of those ES cell markers. Although spontaneous spheroid formation is a process of selective culture of pluripotent stem cells, it may not fully explain the immediate increase in ES cell marker gene expression in spheroids. One possibility is the dedifferentiation of stem (or more differentiated) cells. It has been noted that the spheroid culture condition could restore MSCs to a more primitive status and cause epigenetic changes. For example, it was reported that spheroids from hMSCs showed higher miR-489, miR-370, and miR-433 levels, which play important roles in maintaining the quiescent state of adult stem cells [38–40]. Guo et al. also showed that the change in the histone H3K9 acetylation status changes in spheroids, which may also alter the epigenetic status of spheroid-forming cells [38]. Hypoxia-inducible factor (HIF) is a master transcription factor of hypoxia-associated genes, and HIF-2*α* is reported as one of the factors affecting the pluripotency of MSCs [41]. Although the size of spheroid from CBDCs is relatively small, the inside of spheroids might be hypoxic. This idea was supported by the higher expression of HIF-2*α* shown in this study. The hypoxic condition and the subsequent induction of HIF might be another mechanism that affects the stemness of spheroid-forming cells [4].

In contrast to ES cell markers, the expression of MSC markers is almost identical between spheroid-forming cells and monolayer-cultured cells, which confirmed reports from the previous publications regarding MSCs derived from periodontal ligament cells [37]. One exception was Sca-1, which showed a higher expression in spheroids than monolayer-cultured cells. Sca-1 was originally identified as a marker for hematopoietic stem cells [42, 43], and Sca-1-positive cells are known to have high plasticity, such as the potential to differentiate into cardiomyocytes [44]. Thus, a higher expression of Sca-1 in spontaneously formed spheroids might also reflect a higher plasticity.

In terms of MSCs from bone marrow and adipose tissue, spheroids have been reported to possess enhanced anti-inflammatory, angiogenic, and tissue regenerative effects after transplantation compared with monolayer-cultured cells [45–47]. However, the nature of spheroid-forming cells from MSCs has been investigated only recently, and the information is limited. Furthermore, there was no report on spheroid-forming cells from CBDCs. In parallel with the higher expression of ES cell marker genes in spheroid-forming cells from CBDCs, they showed a higher osteogenic differentiation capability and a higher expression of osteogenic marker genes such as BSP, osterix, and DMP1 than those of monolayer-cultured cells. This phenomenon shows the potential usefulness of spheroid-forming cells from CBDCs for future clinical applications in bone tissue engineering.

In this study, we also investigated the neurogenic differentiation capability of spheroid-derived cells from CBDCs. Immunofluorescence staining showed that the spheroid-derived cells express Nestin and *β*III-tubulin with neuron-like morphology after neurogenic induction, while they are negative in the monolayer-cultured cells. In accordance with the immunofluorescence staining data, the results of qRT-PCR confirmed the higher gene expression of Nestin, MAP2, NGFR, and NeuroD in spheroid-derived cells compared with monolayer-cultured cells. These findings would pave the way for future usage of spheroid-forming cells from CBDCs for neurodegenerative disorders.

Although the results from the current study showed the potential usefulness of spontaneously formed spheroids from CBDCs, there are remaining works toward the clinical application. First, the feasibility of spontaneous spheroid formation should be tested with human cells. Second, the efficiency of spheroid generation needs to be tested. One of the advantages of our protocol is the relatively higher efficiency, since the spontaneous spheroids can be formed from monolayer-cultured cells even after passages. This means a relatively large number of cells are available for spheroid formation, which may allow the production of clinical scale cells from CBDCs. Since spontaneous spheroids possess superior functions compared with monolayer-cultured cells, it might be reasonable to expect a higher homing ability, replication capability, colony-forming efficiency and differentiation capability. Further studies are required to understand the functional aspects of spontaneous spheroids from CBDCs.

Both safety and efficacy are the important issues for clinical application. Although the spontaneous spheroids exhibit ES cell markers, the results from our preliminary in vivo transplantation experiment showed no teratoma formation, which supports the relative safe nature of spontaneous spheroid-derived cells (data not shown). Efficiency of this method with human cells should be confirmed further toward clinical applications.

## 5. Conclusions

Mouse CBDCs can spontaneously form spheroids on a low-adhesion culture plate. The spheroid-forming cells showed a higher gene expression of stem cell marker genes and enhanced osteogenic and neurogenic differentiation capability than cells from conventional monolayer culture systems. Although the direct comparison of spontaneously and mechanically formed spheroids was not performed, our data support the enhanced stemness of spontaneously formed spheroids, thus indicating the usefulness for future clinical applications, such as bone regeneration therapy and treatment of neurodegenerative disorders.

## Figures and Tables

**Table 1:** Immunofluorescence staining antibody reagent list.

Antibody	Dilution	Product no. and manufacturer
*Primary antibodies*		
SSEA1 (mouse monoclonal)	1 : 100	ab16285, Abcam
Sox2 (rabbit polyclonal)	1 : 250	ab97959, Abcam
Oct4 (rabbit polyclonal)	1 : 250	ab19857, Abcam
Nanog (rabbit polyclonal)	1 : 100	ab80892, Abcam
*β*III-tubulin (mouse monoclonal)	1 : 250	ab87087, Abcam
Nestin (mouse monoclonal)	1 : 500	ab6142, Abcam
*Secondary antibodies*		
IgM Alexa Fluor 488 (goat anti-mouse)	1 : 200	ab150121, Abcam
IgG Alexa Fluor 647 (goat anti-rabbit)	1 : 500	ab150079, Abcam
IgG Alexa Fluor 488 (goat anti-mouse)	1 : 500	ab150113, Abcam

**Table 2:** Quantitative reverse transcription-PCR primer set list.

Primer	Direction	Sequence (5′-3′)
*β*-Actin	Forward	CATCCGTAAAGACCTCTATGCCAAC
	Reverse	ATGGAGCCACCGATCCACA

Glyceraldehyde-3-phosphate dehydrogenase (GAPDH)	Forward	GGTGTGAACCACGAGAAA
	Reverse	TGAAGTCGCAGGAGACAA

Sox2/sex determining region Y (SRY)-box 2	Forward	GTTCTAGTGGTACGTTAGGCGCTTC
	Reverse	TCGCCCGGAGTCTAGCTCTAAATA

Fucosyltransferase 4 (FUT4·SSEA1)	Forward	GCAGGGCCCAAGATTAACTGAC
	Reverse	AAGCGCCTGGGCCTAAGAA

Octamer-binding transcription factor 4 (Oct4)	Forward	CAGACCACCATCTGTCGCTTC
	Reverse	AGACTCCACCTCACACGGTTCTC

Nanog	Forward	TGCCAGTGATTTGGAGGTGAA
	Reverse	ATTTCACCTGGTGGAGTCACAGAG

Hypoxia-inducible factors 2*α* (HIF-2*α*)	Forward	CAGTACTCCCACAGGCCTGACTAAC
	Reverse	GACTGTCACACCGCTGCCATA

CD105	Forward	CTGCCAATGCTGTGCGTGAA
	Reverse	GCTGGAGTCGTAGGCCAAGT

CD44	Forward	CAAGCCACTCTGGGATTGGTC
	Reverse	GGCAAGCAATGTCCTACCACAAC

CD29	Forward	CCATGCCAGGGACTGACAGA
	Reverse	GAGCTTGATTCCAATGGTCCAGA

Stem cell antigen-1 (Sca-1)	Forward	TTGCCTTTATAGCCCCTGCT
	Reverse	GTCATGAGCAGCAATCCACA

Kruppel-like factor 4 (KLF4)	Forward	AACATGCCCGGACTTACAAA
	Reverse	TTCAAGGGAATCCTGGTCTTC

Transcription factor Sp7/osterix (OSX)	Forward	AGGCCTTTGCCAGTGCCTA
	Reverse	GCCAGATGGAAGCTGTGAAGA

Bone sialoprotein (BSP)	Forward	GAGACGGCGATAGTTCC
	Reverse	AGTGCCGCTAACTCAA

Dentin matrix protein 1 (DMP1)	Forward	AGTGAGTCATCAGAAGAAAGTCAAGC
	Reverse	CTATACTGGCCTCTGTCGTAGCC

Microtubule-associated protein 2 (MAP2)	Forward	CAGTTTGGCTGAAGGTAGCTGAA
	Reverse	CACATCTGTGTGAGTGTGTGTGGA

Nestin	Forward	GAGGTGTCAAGGTCCAGGATGTC
	Reverse	ACACCGTCTCTAGGGCAGTTACAA

Nerve growth factor receptor (NGFR)/P75NTR	Forward	TCTGATGGAGTCGGGCTAATGTC
	Reverse	CCACAAATGCCCTGTGGCTA

Neuronal differentiation (NeuroD)	Forward	CAAAGCCACGGATCAATCTTC
	Reverse	TGTACGCACAGTGGATTCGTTTC

## Data Availability

The data used to support the findings of this study are included within the article.

## Supplementary Materials

Supplementary Materials The results from flow cytometry of CBDCs for mesenchymal stem cell markers and hematopoietic cell markers. CBDCs at passage 1 were analyzed. CBDCs were positive for mesenchymal stem cell markers including CD29, CD105, CD51, and Sca-1 and negative for CD45 and CD11b.
