# Supplementary material for: Spontaneously Formed Spheroids from Mouse Compact Bone-Derived Cells Retain Highly Potent Stem Cells with Enhanced Differentiation Capability
Source: Stem Cells Int. 2019 May 5;2019:8469012. doi: 10.1155/2019/8469012 (PMC6525826; doi:10.1155/2019/8469012)
Supplement: Supplementary Materials — The results from flow cytometry of CBDCs for mesenchymal stem cell markers and hematopoietic cell markers. CBDCs at passage 1 were analyzed. CBDCs were positive for mesenchymal stem cell markers including CD29, CD105, CD51, and Sca-1 and negative for CD45 and CD11b. [file 8469012.f1.pdf]

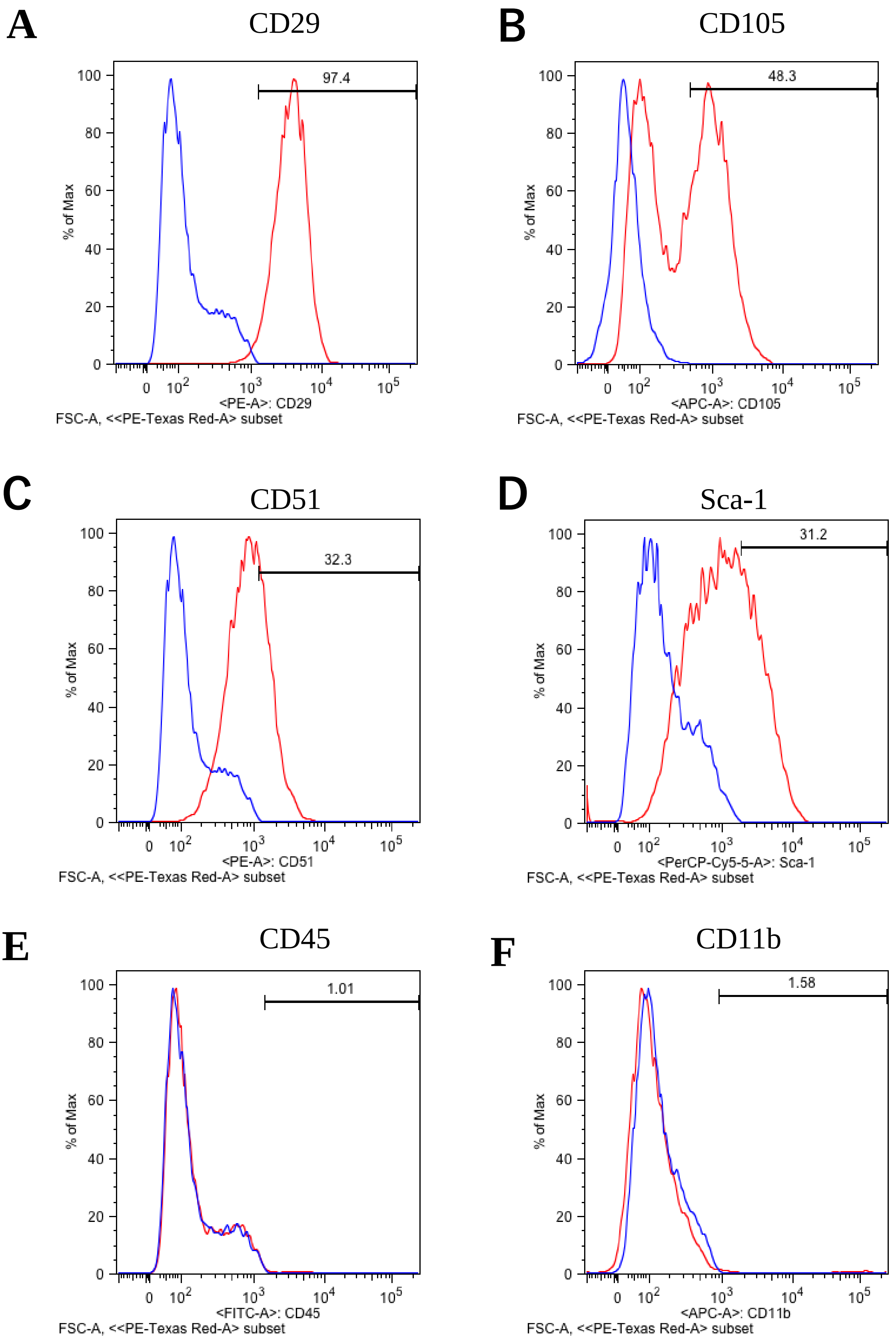

Supplemental figure 1

The results from flow cytometry of CBDCs for mesenchymal stem cell markers and hematopoietic cell markers. CBDCs at passage 1 were analyzed. CBDCs were positive for mesenchymal stem cell markers including CD29, CD105, CD51 and Sca-1 and negative for CD45 and CD11b.
